# Unveiling the genetic basis of Sclerotinia head rot resistance in sunflower

**DOI:** 10.1186/s12870-020-02529-7

**Published:** 2020-07-08

**Authors:** C. V. Filippi, J. E. Zubrzycki, J. A. Di Rienzo, F. J. Quiroz, A. F. Puebla, D. Alvarez, C. A. Maringolo, A. R. Escande, H. E. Hopp, R. A. Heinz, N. B. Paniego, V. V. Lia

**Affiliations:** 1 Instituto de Biotecnología, Centro de Investigaciones en Ciencias Veterinarias y Agronómicas (CICVyA), Instituto Nacional de Tecnología Agropecuaria (INTA); Instituto de Agrobiotecnología y Biología Molecular (IABIMO), INTA-CONICET Nicolas Repetto y Los Reseros s/n (1686), Hurlingham, Buenos Aires, Argentina; 2 Consejo Nacional de Investigaciones Científicas y Técnicas–CONICET, Ciudad Autónoma de Buenos Aires, Argentina; 3 Present address: Biocódices, San Martín, Buenos Aires, Argentina; 4 Facultad de Ciencias Agropecuarias, Universidad Nacional de Córdoba, Ing Agr. Felix Aldo Marrone 746 (5000), Córdoba, Argentina; 5 Estación Experimental Agropecuaria INTA Balcarce, Ruta 226 Km 73.5 (7620), Balcarce, Buenos Aires, Argentina; 6 Estación Experimental Agropecuaria INTA Manfredi, Ruta 9 Km 636 (5988), Manfredi, Córdoba, Argentina; 7 Facultad de Ciencias Exactas y Naturales Universidad de Buenos Aires, Intendente Güiraldes 2160, (1428), Ciudad Autónoma de Buenos Aires, Argentina

**Keywords:** Sunflower, Association mapping, Disease resistance, Argentinian germplasm

## Abstract

**Background:**

*Sclerotinia sclerotiorum* is a necrotrophic fungus that causes Sclerotinia head rot (SHR) in sunflower, with epidemics leading to severe yield losses. In this work, we present an association mapping (AM) approach to investigate the genetic basis of natural resistance to SHR in cultivated sunflower, the fourth most widely grown oilseed crop in the world.

**Results:**

Our association mapping population (AMP), which comprises 135 inbred breeding lines (ILs), was genotyped using 27 candidate genes, a panel of 9 Simple Sequence Repeat (SSR) markers previously associated with SHR resistance via bi-parental mapping, and a set of 384 SNPs located in genes with molecular functions related to stress responses. Moreover, given the complexity of the trait, we evaluated four disease descriptors (i.e, disease incidence, disease severity, area under the disease progress curve for disease incidence, and incubation period). As a result, this work constitutes the most exhaustive AM study of disease resistance in sunflower performed to date.

Mixed linear models accounting for population structure and kinship relatedness were used for the statistical analysis of phenotype-genotype associations, allowing the identification of 13 markers associated with disease reduction. The number of favourable alleles was negatively correlated to disease incidence, disease severity and area under the disease progress curve for disease incidence, whereas it was positevily correlated to the incubation period.

**Conclusions:**

Four of the markers identified here as associated with SHR resistance (HA1848, HaCOI_1, G33 and G34) validate previous research, while other four novel markers (SNP117, SNP136, SNP44, SNP128) were consistently associated with SHR resistance, emerging as promising candidates for marker-assisted breeding. From the germplasm point of view, the five ILs carrying the largest combination of resistance alleles provide a valuable resource for sunflower breeding programs worldwide.

## Background

Sunflower (*Helianthus annuus L*.) is the fourth most widely grown oilseed crop in the world, with an annual production estimated at 36 million metric tons (www.sunflowernsa.com). Unlike other vegetable oils, about 90% of the total production of sunflower oil is used for human consumption, and only 10% is used for biodiesel and industrial applications [1–3].

*Sclerotinia sclerotiorum* causes Sclerotinia head rot (SHR), which is one of the main diseases in sunflower growing areas of Europe, Argentina, and the USA, with epidemics resulting in severe yield losses [4]. This necrotrophic ascomycete poses a considerable problem to agriculture as it attacks more than 400 plant species, including many economically important crops such as rapeseed, soybean, and tomato.

SHR resistance is a complex trait because it is controlled by a polygenic architecture, (i.e. quantitative inheritance with predominance of additive gene action) and has moderate heritability, being greatly affected by different environmental conditions of temperature, humidity, and rainfall, among others [5–9]. During the last two decades, intensive efforts on bi-parental QTL-mapping have led to the identification of ca. 50 quantitative trait loci (QTL) for SHR resistance, with the most robust regions identified in linkage group (LG) 10, followed by LGs 1 and 15 [7, 9–14]. However, the large size of these regions, which often span five or more cM, has precluded the dissection of individual genes, being low resolution capacity one of the main drawbacks of the classical biparental mapping approach. In contrast, association mapping (AM), which relies on the use of a diverse set of germplasm as mapping population, can provide higher-resolution while having the potential to evaluate a wider range of natural variation, without recurrent development of mapping populations [15, 16]. In the earliest implementations of AM, candidate gene approaches, based on prior knowledge of the pathway controlling the trait of interest, dominated the literature. These approaches have been successfully applied to study sunflower resistance to *S. sclerotiorum* in both SHR [17] and stem rot [18]. More recently, new genomic tools made possible the evolution of candidate gene strategies into genomic scans, allowing many SNPs to be queried simultaneously. Although a number of genome-wide association studies resulted in the identification of loci related to flowering time and plant architecture in sunflower [19–21], their power to detect associations for more complex traits, such as quantitative disease resistance, has not been exploited yet. In this context, the use of a larger number of candidate genes by means of medium to high-throughput technologies may serve as a suitable stepping-stone for future large-scale exploration of defense responses to fungal pathogens. In spite of the availability of a reference genome for the species [22], there is still little knowledge about the abundance and distribution of the genes associated with disease resistance. According to Neupane et al. [23], sunflower chromosome 13, followed by chromosomes 9, 4 and 2, contains the highest number of nucleotide Binding Site—Leucine-Rich Repeat (NBS-LRR) genes, which encode disease resistance proteins involved in plant defense .

The goal of the present work was to identify genetic variants associated with SHR resistance. For this purpose, we expanded our initial AM study [17] to include a larger germplasm set, four additional field trials, three additional resistance proxies (i.e. disease severity, area under the disease progress curve for disease incidence and incubation period) and 420 novel stress-related molecular markers.

## Results

### Candidate gene selection, SNP identification and genotyping

Candidate genes (CGs) for SHR resistance were selected from four different data sources (Table 1, see Methods section for details). A total of 27 CGs were suitable for amplification and direct sequencing in the eight sunflower ILs used as a core set (CS) for initial polymorphism discovery. These CGs encompassed 16,441 bp of aligned sequence per individual ( 9,760 bp of coding and 6,681 bp of non-coding regions). The fragment size of each CG ranged from 189 to  1,762 bp including indels. Inspection of Table S1 shows a total of 228 nucleotide changes in the CS, with at least one polymorphic position in 25 of the 27 CGs evaluated. The mean number of SNPs/CG was 8.33, with an average frequency of 1SNP/ 73 bp (excluding indels). The ratio between synonymous (85) and non-synonymous substitutions (50) was 1.7.

**Table 1:** Candidate genes (CGs) for Sclerotinia Head Rot resistance analysed in this study. CGs successfully amplified in the AMP are underlined. ^a^ Acronyms used throughout this article. Gene target, chromosome, Gene start and Gene end refers to the genes annotated in Badouin et al. (2017), available at heliagene.org

Source	Sunflower ID^**a**^	Reference	Fold change	*p*-value	Sequencing primers	Gene target	Gene start (bp)	Gene End (bp)	Chromosome
Sclerotinia differential expression assay (*Helianthus annuus*)	**HeAn_12562**	**Ehrenbolger et al. (2013)** [49]	2.36	0.01	F: 5’AGCCCAGTGTCAAATCAACC3’	AtRLP48	85,297,951	85,299,659	HanXRQChr10
					R: 5′ TCCATACTTACGGTTCCACTCA 3′				
	**HeAn_12761**	**Ehrenbolger et al. (2013) **[49]	0.31	0.001	F: 5’ATGCAAGCATGTGAAGCAGT 3′	PLY2	39,465,262	39,466,985	HanXRQChr17
					R: 5’CCCCAAGCAACATGACTTTT 3′				
	**HeAn_28738**	**Ehrenbolger et al. (2013)** [49]	2.95	0.001	F: 5′ GGTGGACCATTTCATCAAGG 3′	HanXRQChr14g0449241	########	########	HanXRQChr14
					R: 5′ CGTGGGACAGCAACACTAAA 3′				
	**Hean_315**	**Ehrenbolger et al. (2013)** [49]	−2.5	0.052	F: 5′ CCACTCAATCTCCCCAAAAA 3′	HSP41	12,595,842	12,596,417	HanXRQChr06
					R: 5’CACCTTCGCCACTTTAATCC 3′				
	**HeAn_35791**	**Ehrenbolger et al. (2013)** [49]	−2.78	0.001	F: 5’CCGTCACCATTTTCACATTG3′	HanXRQChr13g0415061	########	########	HanXRQChr13
					R: 5’TAAATTTTGGGAACCGGACA3′				
	**HeAn_36589**	**Ehrenbolger et al. (2013)** [49]	−3.7	0.055	F: 5’CTCCGCAACCTCCACTACAT 3’	HSP21	84,168,138	84,168,620	HanXRQChr03
					R: 5’GAGGCAGCTTCTCCACAGTC 3’				
	**HeAn_41021**	**Ehrenbolger et al. (2013)** [49]	12.50	0	F: 5′ CGTCTTCGTTGTTGCAATCT 3′	HanXRQChr16g0506041	39,200,062	39,209,510	HanXRQChr16
					R: 5’AGGAGGAGGATGAGGAGCAT 3′				
	**HeAn_41022**	**Ehrenbolger et al. (2013)** [49]	−4.55	0	F: 5’CAGGCCAAAGTCGAGAATGT 3′	ASF2	39,035,731	39,038,365	HanXRQChr16
					R: 5′ CACAAGGGAAACACAAACACTG 3′				
Sclerotinia differential expression assay (*Brassica napus*)	**G21**	**Zhao et al. (2007) **[34] AT3G17130	18.74	4.49E-5	F: 5′ TGAAGGTCCATCTCCTCTTATTTC 3’	HanXRQChr13g0395221	59,003,751	59,004,311	HanXRQChr13
					R: 5′ GTCGCTAAAGCTCCGTTCAC 3′				
	**G22**	**Zhao et al. (2007)** [34]	18.03	4.82E-6	F: 5′ GGAACCTTATCCACCCGAAT 3′	HanXRQChr06g0170521	17,800,642	17,804,614	HanXRQChr06
		AT4G03400			R: 5′ GCAAATCTTTCTCGGTGTCC 3′				
	**G26**	**Zhao et al. (2007)** [34]	14.92	9.72E-6	F: 5′ CTGATGAGTGGTCGGAGGTT 3′	HanXRQChr10g0292811	95,584,317	95,586,370	HanXRQChr10
		AT3G10190			R: 5′ CCATCATTGTCGGCGTCTA 3′				
	**G28**	**Zhao et al. (2007)** [34] AT1G19100	14.23	8.31E-7	F: 5′ CAGGATAGTAGTCCCGATGGTG 3′	HanXRQChr15g0463011	95,505	102,279	HanXRQChr15
					R: 5′ TCAAAGGTTCCAGTCGCAAA 3′				
	**G30**	**Zhao et al. (2007) **[34]	12.17	1.20E-7	F: 5′ CCCGACACTTATTAAGACTCG 3′	HanXRQChr13g0406751	########	########	HanXRQChr13
		AT5G02140			R: 5′ GCTGATCCTAGTCAACAACTGC 3′				
	**G33**	**Zhao et al. (2007) **[34]	12.16	1.20E-5	F: 5′ TTGCATTATCCTGATGTTATTATCTTG 3′	HanXRQChr16g0509301	62,305,294	62,307,846	HanXRQChr16
		AT3G19630			R: 5′ TTGATGAATTATACCAACCTACCAAA 3′				
	**G34**	**Zhao et al. (2007) **[34]	13.89	3.97E-7	F: 5′ TAGGCGATCGATGCTCACTT 3′	HanXRQChr14g0449241	########	########	HanXRQChr14
		AT3G28390			R: 5′ TCAAGGCGAGTAGGGTAAGC 3′				
Sclerotinia head and stem rot association mapping (*Helianthus annuus*)	**HaGLP3**	**Fusari et al. (2012)** [17]	_	_	F: 5′ TCACATTCTCTTCATCCTATGCTC 3′	HanXRQChr12g0360281	18,742,800	18,743,470	HanXRQChr12
					R: 5′ CCAGTTCCACCAAGAACAGC 3′				
	**HaGLP4**	**Fusari et al. (2012)** [17]	_	_	F: 5′ TGCTTGTAACCTCCTCCTCCT 3′	AB19A	########	########	HanXRQChr10
					R: 5′ TGTGGGAAAAGCATGATGTC 3′				
	**HaGLP5**	**Fusari et al. (2012)** [17]	_	_	F: 5′ TCAAGTGCTATGGCTACTAATGATTT 3′	HanXRQChr06g0181801	74,845,978	74,847,869	HanXRQChr06
					R: 5’TCGATGTATAAATGTATGGTAAAAGAA 3′				
	**HaCOI_1**	**Talukder et al. (2014) **[18]	_	_	F: 5′ CCGATTTGCCACTGGATAAC 3’	COI1	########	########	HanXRQChr14
					R: 5′ ACACGCTGGATAGTCGTTCC 3’				
	**HaCP**	**Fusari et al. (2012)** [17]	_	_	F: 5′ TGCTATTGATGCTGGCAGTT 3′	CYSEP	########	########	HanXRQChr09
					R: 5′ AATCATGTTTCACATACCAAATCTT 3′				
	**HaDRP**	**Fusari et al. (2012)** [17]	_	_	F: 5′ CAAAGGAAGCAACAACACCA 3′	HanXRQChr01g0029501	########	########	HanXRQChr01
					R: 5′ TCGCTGATTCGATACCCTTT 3′				
	**HaPAL**	**Fusari et al. (2012) **[17]	_	_	F: 5′ TGTGGTCTTCAAATTCATTAATAACC 3′	PAL1	62,564,693	62,568,081	HanXRQChr07
					R: 5′ GGCCATTCCTAACAGGATCA 3′				
	**HaTRP**	**Fusari et al. (2012)** [17]	_	_	F: 5′ TTCTTTAGGCCAACCCTCAC 3’	HanXRQChr16g0506311	40,982,842	40,983,492	HanXRQChr16
					R: 5′ CCCTTAATCATAATTCACGAATGTC 3′				
	**HaWP**	**Fusari et al. (2012)** [17]	_	_	F: 5′ CCAAACCCGATGATGATAAA 3′	HanXRQChr10g0289571	69,322,685	69,323,065	HanXRQChr10
					R: 5′ AACAAACAAAACAAGCCACATT 3′				
	**HaWRKY5**	**Fusari et al. (2012) **[17]	_	_	F: 5′ ACACCTCTCAACTTGCACCAAA 3′	HanXRQChr17g0533851	2,017,423	2,019,342	HanXRQChr17
					R: 5′ GCCCATGGATCTGAAGACAAA 3′				
	**HaWRKY7**	**Fusari et al. (2012) **[17]	_	_	F: 5′ CAAGCCGCTAGAGATGGAAC 3′	HanXRQChr16g0509771	66,374,292	66,377,376	HanXRQChr16
					R: 5′ TCAACCCTGTGGTGTTTTGA 3′				
	**HaRhobp_B**	**Fusari et al. (2012)** [17]	_	_	F: 5′ TTGAGGGATTCTAATTGTTATAGTTGA3’	ATROPGEF7	85,118,928	85,122,332	HanXRQChr08
					R: 5′ TTCGGGTGTTCGTCCTTTT 3′				

Twenty of 27 CGs were successfully genotyped in the AMP: eight CGs were genotyped by dHPLC (G22, G26, G30, G34, HaCP, HaDRP, HaPAL, and HaGLP3), two by enzymatic cleavage (CEL1CH) (HeAn_315 and HaGLP5), seven by fluorescent capillary electrophoresis (G33, HaGLP4, HaTRP, HaWP, HaWRKY5, HaWRKY7, and HaRhoBp) and three were typified by direct sequencing (HeAn_41021, HeAn_12562, and HaCOI_1) (Table S1). The number of haplotypes ranged from two to eight, with an average of 3.29 haplotypes per gene.

The 384-SNP Sunflower Oligo Pool Assay (SOPA) matrix retrieved from Filippi et al. [22] was filtered to remove markers that were either monomorphic or showed more than 10% of missing data, yielding a total of 139 bi-allelic SNPs.

Finally, nine SSR previously associated with SHR resistance were successfully amplified in the AMP, ranging from three to eight alleles and with a mean of five (Table S2).

A filter of minor allele frequency (MAF) <0.05 was applied as a final step for CG and marker selection, rendering a total of 168 markers to be tested for association with disease incidence (DI), area under the disease progress curve for disease incidence (AUDPCI), disease severity 168 (DS) and incubation period (IP): 20 CGs, 139 bi-allelic SNPs from the 384-SNPs SOPA and nine SSR. The final data matrix is provided in Table S3.

### Genetic structure, kinship of the AMP-ILs and the relationship with disease response

Bayesian population structure and kinship analysis were based on the 42 SSR used by Filippi et al. [24]. The estimates of population structure for the 135 AMP ILs were similar to those reported by these authors for the original 137 ILs [24]. Again, the AMP is composed of three different genetic groups, with the maintainer/restorer status being the most prevalent characteristic associated with group delimitation (Table S4). The percentage of individuals assigned to a given population, i.e. with inferred ancestry higher than 0.70, was 68.15%.

A genetic distance matrix D, estimated based on the number of shared alleles between all pairs of individuals is presented in Table S5, while the derived hierarchical clustering dendrogram is presented in Fig. S1. Based on the Akaike Information Criteria (AIC), we determined that 60 nested random effects are needed for kinship structure modelling (Fig. S1, red line).

Regarding phenotypic data, the 135 AMP-ILs evaluated for (DI), (AUDPCI), (DS) and (IP) exhibited substantial variability in all disease descriptors, with rankings being similar to those reported by Filippi et al. [24] for the original panel of 137 ILs (Table S4).

**Fig. 1:**
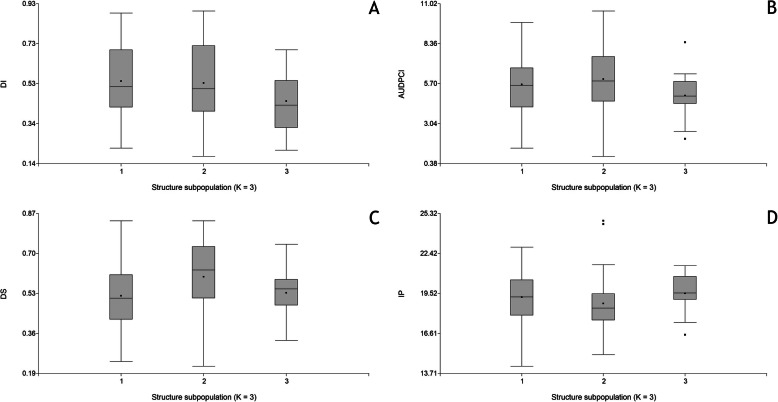
Box-plot of SHR-phenotypic variables in the three STRUCTURE clusters. **a**, DI: Disease incidence; **b**, AUDPCI: Area under the disease progress curve for disease incidence; **c**, DS: Disease severity; **d**, IP: Incubation period

No significant correlation was observed between the genetic distance estimates and the Euclidean distances based on standardised SHR-phenotypic variables (R^2^ = 0.028; Mantel test, *p* = 0.18). Additionally, no significant correlations were observed between ancestries inferred by STRUCTURE and the SHR-phenotypic variables (Table S6). Moreover, visual inspection of the box plots of the adjusted means of SHR-phenotypic variables for the three groups inferred by STRUCTURE revealed no evident differences among groups (Fig. 1), as confirmed by ANOVA (DI, *p* = 0.5771; AUDPCI, *p* = 0.8159; DS, *p* = 0.2428; IP, *p* = 0.7178).

### Association analysis

The four AM models tested were almost equivalent, i.e., they showed similar shape and mean squared differences (MSD) between the observed and expected *p-*values (under the hypothesis of no marker effect) (Fig. S2 and Table S7).

Under the QK model, which includes both the population (Q) and the kinship structures (K), eighty-six markers were associated with SHR resistance before multiple testing corrections (*p* < 0.05) (Table S8). Considering a q-value threshold of 0.2, the number of statistically significant marker-trait associations was reduced to 53 (32 for DI, 6 for AUDPCI, 12 for DS and 3 for IP; *p* < 0.05; *q-value* < 0.2, Table S8). Using a more stringent criterion (*p* ≤ 0.01, *q-value* < 0.20), a total of 23 marker-trait associations were detected (nine for DI, six for AUDPCI, five for DS and three for IP), encompassing 13 different genes (Table 2). The markers SNP117, HaCOI_1, HA1848, and SNP136 were associated with three SHR-phenotypic variables; SNP144 and SNP128 were related to two variables; while G33, G34, SNP125, SNP80, SNP60, SNP23, and SNP44 were associated with a single variable. The percentage of variation explained by each marker was 13.98% on average, ranging from 6.79 to 25.31% (Table S8). The markers SNP117, HaCOI_1, and HA1848 showed the most significant association with disease resistance (*p* < 0.01, *q*-*value* < 0.10).

**Table 2:** Markers associated with SHR resistance (*p* ≤0.01; *q-value* < 0.20). Physical mapping of the associated markers, as well as Molecular Function and Gene Onthology (GO) assignment were retrieved from Heliagene.org, using the genome XRQ v1.0 (Badouin et al. 2017) [22]

Marker	Chromosome	Position (bp)	Origin	Phenotypic variable	Molecular function	GOs
SNP117	10	132,349,611	Illumina SNP-array	DI, AUDPCI, IP	3-oxo-Delta(4,5)-steroid 5-beta-reductase	P:cellular metabolic process; F:coenzyme binding; F:catalytic activity
HaCOI_1	14	168,171,427	CG (Talukder et al. 2014) [18]	DS, AUDPCI, IP	insensitivity to coronatine	F: protein binding
HA1848	7	–	SSR (Zubrzycki et al. 2017) [9]	DS, DI, AUDPCI	–	–
SNP136	9	72,028,226	Illumina SNP-array	DS, DI, AUDPCI	Beta-hexosaminidase 1	P:carbohydrate metabolic process; C:cytoplasmic membrane-bounded vesicle; F:beta-N-acetylhexosaminidase activity; F:cation binding
SNP144	13	14,745,225	Illumina SNP-array	DI, AUDPCI	Tyrosine transaminase family protein	F:pyridoxal phosphate binding; P:cellular amino acid and derivative metabolic process; F:1-aminocyclopropane-1-carboxylate synthase activity; P:vitamin E biosynthetic process; F:L-tyrosine:2-oxoglutarate aminotransferase activity
SNP128	12	18,758,084	Illumina SNP-array	DI, AUDPCI	Germin-Like Protein 3 (HaGLP3)	F:superoxide dismutase activity; C:plant-type cell wall; F:oxalate oxidase activity; F:nutrient reservoir activity; P:oxidation reduction; P:auxin-mediated signaling pathway; F:oxidoreductase activity;
G33	16	62,306,954	CG (Zhao et al. 2007) [34]	DS	Radical SAM superfamily protein	F: catalytic activity; iron-sulfur cluster binding
SNP125	2	120,750,653	Illumina SNP-array	DI	Xylulose kinase-2	phosphate metabolic process;transferase activity, transferring phosphorus-containing groups;phosphotransferase activity, alcohol group as acceptor;primary metabolic process;carbohydrate metabolic process
SNP80	14	170,322,057	Illumina SNP-array	DI	Embryonic flower 2	P:genetic imprinting; P:negative regulation of flower development; F:transcription factor activity; C:nucleus
SNP60	10	173,066,810	Illumina SNP-array	DI	Serine-threonine/tyrosine-protein kinase	P: protein phosphorylation, F: Protein- serine/threonine kinase activity, F: ATP binding.
G34	14	137,657,256	CG (Zhao et al. 2007) [34]	DI	ATP-binding cassette, subfamily C, member 9	P: potassium ion transport; F: ATP binding; sulfonylurea receptor activity; C: membrane
SNP23	15	16,332,782	Illumina SNP-array	IP	Rho-associated protein kinase 1/2	P:protein amino acid phosphorylation; F:ATP binding; F:protein serine/threonine kinase activity
SNP44	6	52,738,650	Illumina SNP-array	DS	GRAS family transcription factor	P: positive regulation of transcription, DNA-templated regulation of transcription, DNA-templated, response to chitin, response to xenobiotic stimulus; F: DNA-binding transcription factor activity, sequence-specific DNA binding

*DI* disease incidence, *DS* disease severity, *AUDPCI* area under the disease progress curve for disease incidence and *IP* incubation period

The 13 associated genes (*p* ≤ 0.01, *q-value* < 0.2) were mapped against the sunflower reference genome and localised on chromosomes 2, 6, 7, 9, 10, 14, and 15. The GO-terms of the genes harbouring the associated markers are detailed in Table 2. In addition, we also surveyed the annotation of all the genes located ±1 Mb flanking regions upstream and downstream from all the associated markers, in order to build a catalog of all genes that could be involved in SHR resistance. A total of 124 genes were identified within these regions, which ranged from one to 39 per associated marker. Location, description and distance to the associated marker are given in Table S9.

To investigate the patterns of variation in the associated markers, we ranked the ILs according to the number of favourable alleles (Table S4). The top four ILs were 51084/C820, RHA801, HA441, and 51084, with the number of favourable alleles ranging from eleven to nine (Table 3). For these ILs, the adjusted means of DI, DS and AUDPCI were within the first quartile in all cases (with the exception of IL 51084/C820 for DI and ILs 51084/C820 and RHA801 for DS), while the IP values fell within the last quartile, i.e. the most resistant. These ILs also showed sustained levels of resistance across field trials. In agreement with these findings, significant and negative correlations were found between DI, AUDPCI, DS and the number of favourable alleles (Spearman, *p* < 0.01, Table S10), while a positive correlation was observed for IP and the number of favourable alleles (Spearman, *p* < 0.01, Table S10).

**Table 3:** Performance of the inbred lines with the highest number of favourable alleles

Inbred Line	SNP117	HA1848	SNP136	SNP128	SNP144	SNP125	SNP80	G34	SNP60	HaCOI_1	G33	SNP44	SNP23	DI	AUDPCI	DS	IP
**51084**	**x**		**x**		**x**		**x**	**x**	**x**	**x**	**x**	**x**		**32.1**	**3.81**	**45.5**	**19.88**
**51084/C820**	**x**	**x**	**x**		**x**	**x**	**x**	**x**	**x**		**x**	**x**	**x**	**46.2**	**4.03**	**49.4**	**21.20**
**RHA441**			**x**	**x**	**x**	**x**	**x**	**x**		**x**			**x**	**21.8**	**2.52**	**32.9**	**21.53**
**RHA801**	**x**			**x**	**x**	**x**	**x**		**x**	**x**			**x**	**31.8**	**3.75**	**50.5**	**20.79**

*DI* disease incidence, *DS* disease severity, *AUDPCI* area under the disease progress curve for disease incidence and *IP* incubation period

## Discussion

*Sclerotinia sclerotiorum* resistance is a complex trait with medium to low heritability and a largely unknown genetic basis*.* In this work, an AM study encompassing 135 sunflower ILs was conducted to identify genes involved in SHR resistance. Despite the increasing attention being paid to genome-wide approaches, CG AM remains a robust strategy to explore phenotype-genotype associations. Careful selection of CGs can greatly improve success rates, particularly when there is little knowledge about the molecular mechanisms underlying the trait of interest. Here, we detected 13 markers associated with SHR resistance using different sources of information as starting points. Unlike previous AM approaches to SHR, that investigated from eight to sixteen CGs [17, 18], here we evaluated 27 CGs, a panel of nine SSRs previously associated with SHR resistance via bi-parental mapping and a set of 384 SNPs located in genes related to biotic and abiotic stress responses, such as hypersensitive response, jasmonic-acid and auxin mediated signaling pathways, response to oxidative stress, calcium associated genes, germin-like proteins, among others [9]. Moreover, the complexity of the trait led us to score four disease descriptors, making this study the most exhaustive AM approach to disease resistance in sunflower to date.

An understanding of the effect of population structure on the trait of interest is critical to avoid spurious associations in AM studies. If population structure accounts for too much of the variation, then structured association analyses will have low power to detect the effects of individual markers [25]. In our AMP, correlations were found neither between the Euclidean distances based on SHR-phenotypic variables and the matrix of genetic distances, nor between individual variables and the assignment scores from Bayesian analyses. This suggests that population structure would have little impact on the detection of marker-trait associations.

Another key aspect in AM mapping studies is the inclusion a measure of relatedness among individuals within and between subpopulations, as coancestry coefficients calculated from pedigree records or marker-based kinship estimates [26]. The kinship structure implies a correlation structure of observations in the framework of a statistical model. Modelling kinship structure as a set of nested random factors, as we did here, is an alternative approach to the inclusion of the kinship matrix as a correlation matrix of an IL random effect. This approach allows the use of standard statistical software for mixed model fitting.

To test for associations, we first applied a naïve approach that did not include any correction for the level of relatedness or structure between ILs (model **SM**). The curves of observed vs. expected *p*-values (under the hypothesis of no marker effect) and the mean square difference between them [26], showed that accounting for population structure (model **Q**) did not contribute to a clear reduction in the number of significant marker-trait associations (*p* < 0.05). The same result was obtained by including the kinship structure (model **K**) and both sources of structure (model **QK**). These results are in accordance with the fact that none of the SHR-phenotypic variables showed association with the underlying population structure, thus minimising the risk of spurious associations derived from marker-trait covariance [27].

A *q*-value was calculated for the entire set of *p*-*values* to establish a reasonable false discovery rate [28, 29]. Three markers, HA1848, HaCOI_I and SNP117, located in chromosomes 7, 10 and 14, respectively, had the most significant associations with disease resistance (*p* < 0.01; *q*-*value* < 0.10). Moreover, each of these markers were associated with three of the four SHR phenotypic variables. Two of these associated markers validate a previous biparental mapping study of *Sclerotinia* resistance in sunflower [9]. The SSR marker HA1848 was reported to co-localise with a QTL for decrease in SHR DI [9], while the CG HaCOI_1 appeared to be related to *Sclerotinia* stem rot resistance [18]. The HaCOI_1 CG is orthologous to the *Arabidopsis thaliana* COI1 gene, which encodes a leucine-rich repeat (LRR) protein essential for the Jasmonic acid (JA) signaling pathway. The JA has a crucial role in plant defense against biotic and abiotic stresses [30]. Indeed, necrotrophic fungal pathogens, like *S. sclerotiorum*, are known to act as primary activators of JA-dependent defenses through the COI1 receptor [31].

The gene harbouring SNP117 was assigned to the GO category *progesterone 5β-reductase (P5βR)*. In spite of being a family first described in animals, PRISEs (progesterone 5*β*-reductase and/or iridoid synthase-like 1,4-enone reductases) are ubiquitous in plants and are involved in cardenolide and iridoid biosynthesis in many species [32]. The occurrence of species with these enzymes but not producing cardenolides or iridoids may indicate a more general role in the scavenging and detoxification of reactive carbonyl compounds [32]. In agreement with this proposal, members of this family showed to be sensitive to abiotic and mechanical stressors, with their expression being regulated by ethylene and hydrogen peroxide [33].

At a less stringent threshold (*p* ≤ 0.01; 0.10 < *q-value* < 0.20), ten additional markers, distributed across eight different chromosomes were associated with resistance. The orthologs of two of these genes, G33 and G34, were previously identified as differentially expressed in response to *Sclerotinia* infection in *Brassica napus* [34]. G34 encodes an ABC transporter and has several known functions of agricultural importance, with detoxification and auxin transport being one of the most important [35]. Auxing mediated signaling is not only related to plant grow and development, but also to pathogen resistance, mainly for necrothophic fungi [36, 37].

G33 encodes a member of the Radical SAM Superfamily, which contains over 100,000 homologous enzymes that catalyze a broad range of reactions required for life [38], including disease resistance [39].

The remaining eight markers include a germin-like protein (GLP, SNP128), a β-hexosaminidase (SNP136), a putative rho-associated protein kinase (SNP23), and five genes with diverse annotations (i.e. Tyrosine transaminase, Embryonic flower 2, Xylulose kinase, Serine-threonine/tyrosine kinase, GRAS family transcription factor). Germins and GLPs are encoded by gene families with multiple members, and a wealth of evidence supports their involvement in plant defense. Many germins possess oxalate oxidase (OXO) activity, i.e. can degrade oxalic acid to H_2_O_2_ and CO_2_ [40, 41], whereas GLPs show superoxide dismutase (SOD), phosphodiesterase, polyphenol oxidase, protease inhibition or proteolytic activities [42]. In particular for *S. sclerotiorum*, Rietz et al. [43] reported the role of GLPs in the defense response of *B. napus* against the pathogen. More recently, overexpression of sunflower HaGLP1 in *A. thaliana* was also shown to promote reactive oxygen species (ROS) accumulation and enhance protection against *S. sclerotiorum* and *Rhizoctonia solani* [44].

The marker SNP136 is of particular interest since it falls within a *β*-n-acetylglucosaminidase that catalyses the hydrolysis of chitin oligosaccharides. Chitin (*β*-1,4-linked polymer of N-acetylglucosamine) is commonly present in fungal cell walls, and its fragments are known to act as potent elicitor signals in plant defense [45].

Fusari et al. [17] found that one allele of a putative Rop-interactive CRIB motif-containing protein (rhoBP_B//RIC) was associated with a decrease in SHR DI. This association could not be assessed in our AMP, because the beneficial allele was at a very low frequency (< 0.05). However, we were able to identify another putative Rho-associated protein kinase related to an increase in SHR resistance (SNP23), further suggesting that this family plays a role in the defense process.

The causal polymorphism for a QTL can be distant from the marker under scrutiny, particularly in species with high levels of linkage disequilibrium, such as sunflower [20, 46, 47]. In line with this notion, several of the associated markers found in our work show allelic differences leading to synonymous substitutions. Mining of the recently available sunflower genome [22] identified a considerable number of promising candidates for future evaluation. Genes related to defense processes located in the vicinity of the associated markers include a protein phosphatase 2C, nucleotide binding site–leucine-rich repeat (NBS-LRR) R proteins, acetyltransferases, GRAS proteins and various GLPs. In sum, we generated a catalog of 124 candidate genes, putatively involved in the SHR resistance process.

Overall, we found that most of the genes associated with resistance to SHR have molecular functions involving the accumulation and degradation of ROS and JA-mediated signaling pathways, which are primary processes in plant-pathogen interactions. In agreement with these findings, Na et al. [48] reported high transcript abundance for marker genes involved in both the SA and JA pathways in a resistant sunflower cultivar challenged with the pathogen *S. sclerotiorum*. In addition, H_2_O_2_ levels and ROS scavenger enzyme activities were also increased, as well as callose deposition on the cell wall and soluble protein content.

As genotyping costs continue to decrease, phenotyping efforts are becoming the most critical issue for AM approaches. Although high correlations were observed among the four examined phenotypic variables [6], there were several associations with only one disease descriptor, suggesting that, despite some overlapping, all of them give insights into different aspects of resistance. In agreement with the results of the statistical analysis, the distribution of the favourable alleles of the associated markers among the ILs of the AMP is consistent with their pedigree and prior behaviour in field trials. Significant and negative correlations were found between DI, AUDPCI, DS and the number of favourable alleles, while a positive correlation was observed for IP and the number of favourable alleles, suggesting that ILs with a higher number of favourable alleles tend to have lower DI, AUDPCI and DS while longest IP. Indeed, the highest number of favourable allelles corresponded to ILs showing a stable response across growing seasons [6] and ranked among the most resistant in terms of DI, DS, AUDPCI and IP values. Moreover, recent field observations indicate that these ILs also have enhanced resistance to head and stem canker caused by *Phomopsis heliantii* (Corro Molas A., pers. comm.). In sum, our results highlight that the adoption and application of strategies combining a moderate number of markers with previuos genomic information, as CG-association mapping, allows the identification of strong genomic signals associated with traits of agronomic importance, even for low heritability traits, as SHR resistamce. In this regard, this work brings the first hints towards the characterization of sunflower resistant genes to SHR, while contributing to marker-assisted breeding for crop improvement.

## Conclusions

Four of the SHR resistance-associated markers identified in this work, HA1848, HaCOI_1, G33 and G34, validate previous research and emerge as promising candidates for marker-assisted breeding. Moreover, our work allowed the identification of novel markers, i.e. SNP117, SNP136, SNP144 and SNP128, consistently associated with SHR resistance, as determined by using different disease descriptors. Further studies are currently under way to confirm their effect on resistance using mutagenised populations. From a germplasm perspective, the ILs with the largest combination of resistance alleles also provide a valuable resource for sunflower breeding programs worldwide.

## Methods

### Plant material and sources of phenotypic data

The development of the sunflower association mapping population (AMP) used here is described in Filippi et al. [24]. Briefly, a diverse collection of 137 cultivated sunflower inbred lines (ILs) was selected by the sunflower breeders of the Instituto Nacional de Tecnología Agropecuaria of Argentina (INTA) and preserved at the Active Germplasm Bank of INTA Manfredi (BAG-IM). All the ILs are breeding resources obtained under institutional and national guidelines (see Filippi et al. [24] for details on the original source of plant materials). Genetic diversity analyses of these ILs using 42 SSR markers, as well as a panel of 384 SNPs, indicated that our AMP exhibits a high percentage of the allelic diversity present within the cultivated sunflower gene pool [24].

The susceptibility of the original 137 AMP ILs towards *S. sclerotiorum* was assessed by Filippi et al. [6] in five consecutive field trials (FTs) conducted at the Balcarce Experimental Station INTA (37° 50′ 0″ S, 58° 15′ 33″ W, Province of Buenos Aires, Argentina) from 2009 to 2014. The FTs were conducted in a randomized complete block design with two blocks. The variables used as a proxy for SHR resistance were disease incidence (DI), disease severity (DS), area under the disease progress curve for disease incidence (AUDPCI) and incubation period (IP).

A full description of the experimental field design, fungal isolation, inoculum preparation and statistical methods associated to adjusted mean estimation for all the SHR-phenotypic variables can be found in Filippi et al. [6].

In the present study, the number of AMP ILs was reduced to 135 because two of them were contaminated, so both genetic and phenotypic statistical analyses were repeated in order to obtain specific values for this reduced IL population. Seeds were provided by the Active Germplasm Bank of INTA Manfredi.

### DNA extraction and genotyping

Total DNA was extracted from leaf tissue from three individuals of each IL using NucleoSpin Plant II kit (Macherey-Nagel, Germany), following manufacturer’s instructions. The quality and concentration of the genomic DNA were assessed using electrophoretic analysis and Picogreen® technology (Invitrogen, San Diego, CA).

Three types of molecular markers were used to test for association with SHR resistance: 1) PCR amplicons: a panel of 27 candidate genes (CGs) selected from Zhao et al. [34], Ehrenbolger et al. [49], Fusari et al. [17] and Talukder et al. [18] (Table 1); 2) SNPs: 384 SNPs from a custom Illumina Oligo Pool Assay designed on the basis of stress-related candidate genes (Sunflower Oligo Pool Assay, SOPA, [9, 24]); and 3) SSRs: a set of 9 SSR markers recently associated with SHR resistance by bi-parental QTL mapping in sunflower [9].

#### PCR amplicons

Table 1 provides general information on the CGs and the primers used for amplification. For CGs selected from a transcriptional analysis of *A. thaliana* in response to *S. sclerotiorum* [34], we first used the phylogenetic approach of Fusari et al. [17] to identify the corresponding orthologous genes in sunflower. We made an initial step of polymorphism assessment for all the CGs because no knowledge of diversity levels was available. Direct Sanger sequencing of the 27 CGs was performed in a core set (CS) of eight inbred lines to identify haplotypes and select the best genotyping method for the AMP. The simulated annealing algorithm, implemented in the software PowerMarker V. 3.51 [50] (Liu & Muse 2005), was used for CS construction, with 1000 replicate analyses [51]. The CS included ILs 2091, 2121, 51084/5429, B99, C154B, C625U, R419 and R449. CGs with only two haplotypes were genotyped using denaturing High-Pressure Liquid Chromatography (dHPLC) or digestion by CEL1 endonuclease, as described in Fusari et al. [52]. Haplotypes differing in fragment size were genotyped by fluorescent capillary electrophoresis (FCE). Finally, CGs with more than two haplotypes were genotyped by direct sequencing. All sequencing reactions and analyses were performed as described in Fusari et al. [46].

#### Sunflower Oligo Pool assay (SOPA)

The 384 Illumina SOPA was designed to target SNPs selected by *in-silico* searches of EST-databases focusing on genomic regions with molecular functions related to stress responses [9]. Among the initial panel of 384 SNPs on the array, 182 were found to be variable in our AMP [24], with 139 passing the minor allele frequency filters used for this study. SNP polymorphism data for the AMP are available in Filippi et al. [24].

#### SSR markers

A total of nine SSR markers were selected from a study of QTL mapping for SHR resistance on a biparental sunflower population derived from the cross of the public French ILs PAC2 x RHA266 [9]. Genotyping was carried out as described in Filippi et al. [24]. The general features of the SSR markers are given in Table S2.

### Population structure and relatedness

As mentioned before, due to the reduction in the number of ILs compared to our previous analyses, population structure was re-estimated using the Bayesian approach implemented in STRUCTURE [53] based on the 42 SSR described in Filippi et al. [24]. Two of these SSR (HA77 and HA1848), were also tested for phenotype-genotype associations.

The kinship structure (*K*) was modelled as a set of nested random effects. In this regard, a matrix (S) of proportion of shared alleles was generated from the above-mentioned 42 SSR and used as input for the estimation of the distance matrix $D=\sqrt{1-S}$. The agglomerative hierarquical cluster was generated with the *ward. D* agglomeration method. The resulting dendrogram was cut at different levels (nodes) and as a result, ILs were grouped into 2 to 134 clusters. The Akaike Information Criteria was used to determine the optimal number of grouping factors needed to model the kinship structure.

To assess the relationship between disease response and the genetic structure of the AMP, we carried out a Mantel test comparing an Euclidean distance matrix based on the standardised adjusted means of the phenotypic variables with the genetic distance matrix D. In addition, we conducted a correlation analysis between disease response variables and the STRUCTURE ancestry coefficients of each inbred line. Finally, one-way ANOVA was used to test for significant differences in disease response variables among STRUCTURE clusters; ILs were assigned to a given cluster when inferred ancestry was > 0.70.

### Association mapping

Statistical analyses were performed according to the two-step method described in Stich et al. [26]. Briefly, it aims at obtaining adjusted means for each IL, considering the effects of year, inoculation date and the plot structure of the experimental design. In the second step, the adjusted mean derived from the first step is used as the response variable and is fitted for one marker at a time. We considered the four following models:

1. A simple linear model (**SM)**, which only includes a fixed effect for the marker (*M*_*i*_), under the assumption of independence of errors.

$$ {Y}_{ij}=\mu +{M}_i+{e}_{ij} $$

2) The **Q** model, which extends the Generalised Linear Model (GLM) by including the population structure as a set of regressor variables corresponding to the STRUCTURE ancestry assignment matrix ***Q*.**

$$ {Y}_{ij}=\mu +{M}_i+ Q\beta +{e}_{ij} $$

3) The **K model**, which does not include population structure but a set of nested random factors represented by the term **Zk**, where ***Z*** is an incidence matrix and ***k*** is a random effects vector (see the representation of the kinship structure in the previous section).

$$ {Y}_{ij}=\mu +{M}_i+ Zk+{e}_{ij} $$

4) The model **QK**, which includes both the population and the kinship structures.

$$ {Y}_{ij}=\mu +{M}_i+ Q\beta + Zk+{e}_{ij} $$

Correlations among SHR-phenotypic variables and the number of favourable alleles were estimated using Spearman’s rank correlation.

The R environment [54] was used for most of the statistical analyses. Library *lme4* [55] was used for model fitting. The function *hclust* with method *ward.D* was applied for cluster analysis, while the function *cutree* was implemented to cut the dendrogram that generated the grouping factors used to model kinship. Complementary statistical analyses were performed by means of InfoStat [56] and InfoGen [57].

The false discovery rate (FDR) thresholds were determined using the QVALUE package [58] and a *q-value* cut-off of 0.2, as suggested by Iquira et al. [28].

### Annotation and genomic context of the associated markers

Given that most of our SNP markers were located within CGs, we retrieved the annotations of the genes harbouring the associated markers using BLASTN searches against the reference genome of *Helianthus annuus* (available at http://www.heliagene.org). We also searched for the genes surrounding the associated markers. To ensure physical linkage [47], we scanned up to ±1 megabase from the marker of interest (XRQ version 1.0, [22]).

## Supplementary information

**Additional file 1: Table S1.** Diversity estimates of candidate genes (CGs) for Sclerotinia Head Rot resistance.

**Additional file 2: Table S2.** SSR markers that co-localised with QTLs for Sclerotinia Head Rot resistance (Zubrzycki et al. 2017).

**Additional file 3: Table S3.** Genotypic data used for association mapping.

**Additional file 4: Table S4.** Adjusted means across five field trials for Sclerotinia Head Rot-related phenotypic variables, STRUCTURE ancestry coefficients (K = 3) and number of favourable alleles for the inbred lines of the Association Mapping Population (AMP).

**Additional file 5: Table S5.** Phenotypic and genetic distance matrices for the 135 Inbred lines (ILs).

**Additional file 6: Table S6.** Spearman correlations among Sclerotinia Head Rot (SHR)-phenotypic variables and the ancestry groups inferred by STRUCTURE.

**Additional file 7: Table S7.** Mean squared differences (MSD) between observed and expected *p*-values for the four association models applied in the study.

**Additional file 8: Table S8.** Results of the association mapping analysis. Statistical significance and mean ± SE (standard error) per Sclerotinia Head Rot (SHR)-related phenotypic variable and genotype.

**Additional file 9: Table S9.** Putative genes in the vicinity of the associated markers.

**Additional file 10: Table S10.** Spearman correlations among Sclerotinia Head Rot (SHR)-phenotypic variables and the number of favourable alleles in each inbred line.

**Additional file 11: Figure S1.** Hierarchical clustering dendrogram based on genetic distance between the 135 sunflower inbred lines.

**Additional file 12: Figure S2.** Plot of the observed vs expected p-values for the four association models studied for all traits.

## Data Availability

All data generated or analysed during this study are included in this published article [and its supplementary information files]. Sequence Data is available at GenBank: MT593377 to MT593867. Additional information related to intermediate statistical processing steps is available upon request.

## Abbreviations

AMP: Association mapping population
AUDPCI: Area area under the disease progress curve for disease incidence
CGs: Candidate genes
CS: Core set
DI: Disease incidence
DS: Disease severity
FCE: fluorescent capillary electrophoresis
ILs: Inbred lines
IP: Incubation period
SHR: Sclerotinia head rot
SOPA: Sunflower Oligo Pool Assay
