# Supplementary figures and images for: Unveiling the genetic basis of Sclerotinia head rot resistance in sunflower
Source: BMC Plant Biol. 2020 Jul 8;20:322. doi: 10.1186/s12870-020-02529-7 (PMC7346337; doi:10.1186/s12870-020-02529-7)

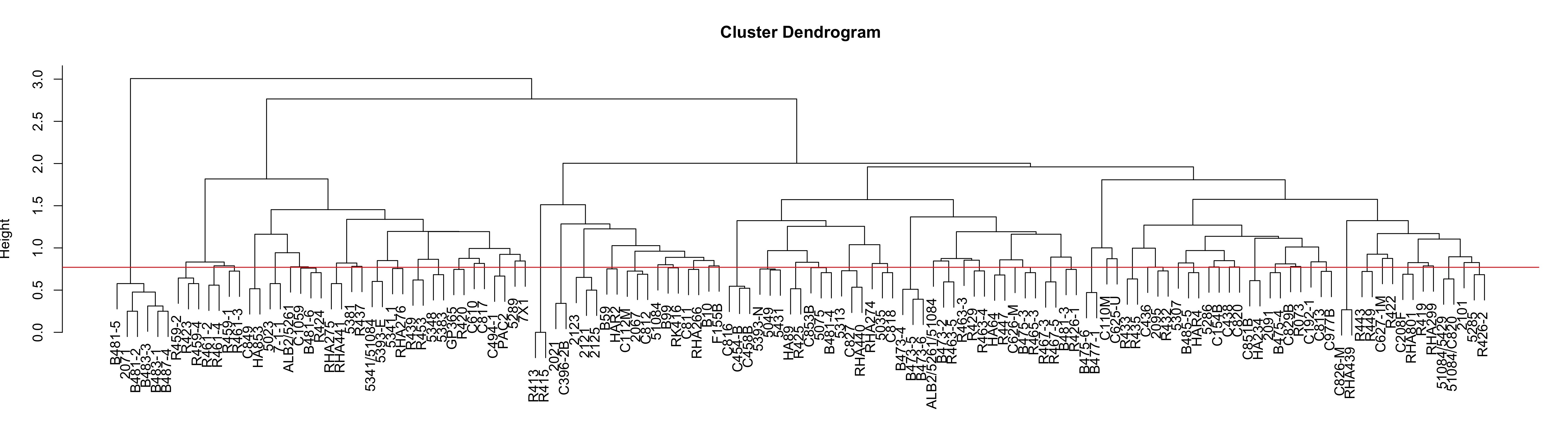

Supplement: Supplementary file 11 — Additional file 11: Figure S1. Hierarchical clustering dendrogram based on genetic distance between the 135 sunflower inbred lines. [file 12870_2020_2529_MOESM11_ESM.jpg]

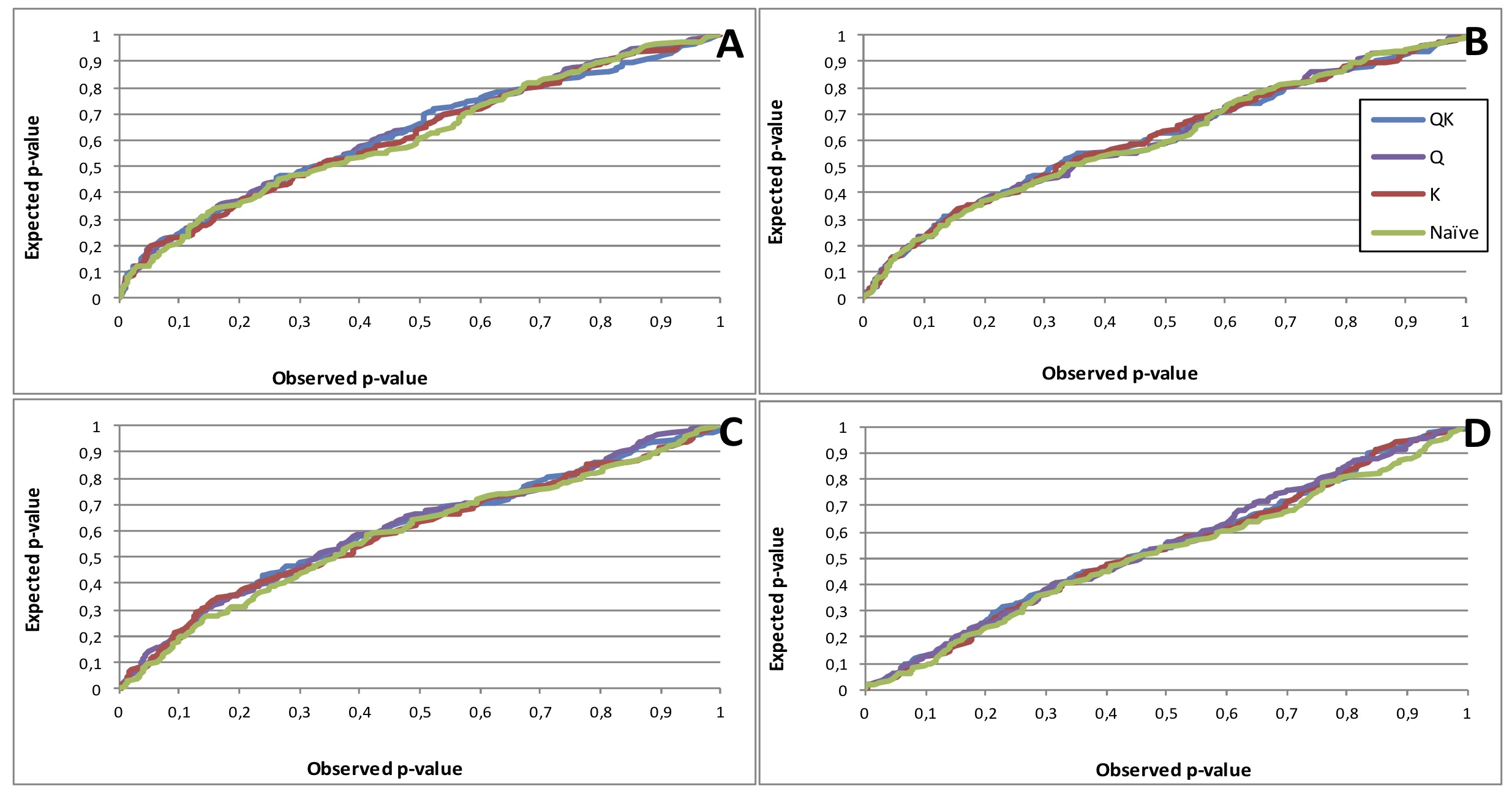

Supplement: Supplementary file 12 — Additional file 12: Figure S2. Plot of the observed vs expected p-values for the four association models studied for all traits. [file 12870_2020_2529_MOESM12_ESM.jpg]
